# True *Dicrocoelium* Spp. Infection in an Immigrant Traveler (VFR)

**DOI:** 10.4269/ajtmh.20-1354

**Published:** 2021-03-15

**Authors:** Carmen Lavilla-Salgado, Cristina Carranza-Rodríguez, José-Luis Pérez-Arellano

**Affiliations:** 1 Unit of Infectious Diseases and Tropical Medicine, Insular Universitary Hospital of Gran Canaria, Las Palmas de Gran Canaria, Spain;; 2 Department of Clinical and Surgical Sciences, University of Las Palmas Gran Canaria, Las Palmas de Gran Canaria, Spain

## CLINICAL REPORT

**Figure 1.**
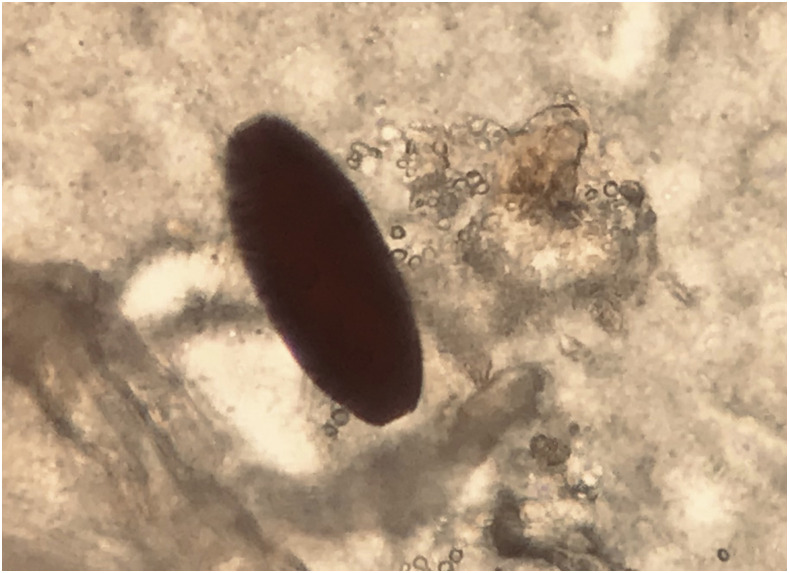
Embryonated egg of *Dicrocoelium* spp. in fecal sample. They have an asymmetrical oval shape and measure approximately 40 × 25 µm, are of dark brown color, and have a smooth thick shell and an indistinct operculum. This figure appears in color at www.ajtmh.org.

A 57-year-old man, born in Ghana but residing in the Canary Islands (Spain) for 30 years, was attended to in our Tropical Medicine Unit after he returned from a month long trip to his country of origin to visit relatives. He complained of diarrhea of 5 days’ duration, with 4–5 stools daily, and the presence of blood and mucus in the stools. In addition, he presented with colicky abdominal pain in the right hypochondrium, without nausea, vomiting, fever, or chills. During his stay in Ghana, the patient had eaten food prepared in poor sanitary conditions. On physical examination, deep palpation of the right hypochondrium elicited a mild pain. No skin or mucosal involvement, peripheral lymphadenopathy, and spleen or liver enlargement was observed. Plain chest radiography and abdominal ultrasound were normal. Complete blood count and chemistry panel revealed eosinophilia (1,700 eosinophils/µL) but no other abnormalities. His stool samples were all examined fresh, by direct saline smear, followed by the formol-ether concentration method. In concentrated samples, we found several embryonated helminth eggs, with morphological characteristics suggestive of *Dicrocoelium* spp. (Figure 1).

After 3 days of controlled food intake, the parasitological stool study was repeated, and the presence of *Dicrocoelium* spp. eggs was confirmed. It was therefore considered a true infection and treated with praziquantel (25 mg/kg/8 hours for 1 day). The patient evolved favorably, the symptoms disappeared, and eosinophilia decreased to normal values (100 eosinophils/µL). Repeated coproparasitic studies at 2-week intervals continued to be negative. The study was completed with magnetic resonance cholangiopancreatography, which ruled out the presence of parasites in the bile duct.

*Dicrocoelium dendriticum* is a trematode found worldwide that tends to live in the bile ducts of herbivorous livestock. In Ghana, *Dicrocoelium hospes* is a common parasite of cattle, and it is suggested that true infections in man may also occur.^1^ This helminth has a complex biological cycle because it requires two intermediate hosts (snails and ants) to complete its development.^2^ Exceptionally, humans can become definitive hosts after accidental ingestion of infected ants or food contaminated with them (true parasitism). This situation should be differentiated from pseudo-parasitism, which corresponds to the detection of eggs in feces due to the ingestion of the liver from parasitized animals^3^ (Table 1). Repeating the parasitological stool examination after a viscera-free diet for 3 days allows us to differentiate the two situations.^3^

**Table 1:** Differences between true and pseudo-parasitism *Dicrocoelium* spp. infection

	True parasitic disease	Pseudo-parasitism
Clinical manifestations	Yes	No
Eosinophilia/elevated IgE	Yes	No
Impaired liver tests/pancreatic	Possible	No
Egg characteristics	Embryonated	“In transit”
Egg elimination after 3 days of controlled diet	Yes	No
Praziquantel treatment	Yes	No

There are just a few published cases of true parasitization with *Dicrocoelium* spp. in humans, in different parts of the world.^2–4^ In such cases, infection may be asymptomatic or it may manifest with pain, abdominal distension especially in the right hypochondrium, diarrhea or constipation, vomiting, eosinophilia, a slight increase in transaminase levels, and hepatomegaly.^3,4^ Exceptionally, cases of biliary obstruction and cholangitis have also been described.^5^ True cases can be treated with praziquantel or triclabendazole, which is unnecessary in pseudo-parasitism infection.^3^
